# Predicting opioid receptor binding affinity of pharmacologically unclassified designer substances using molecular docking

**DOI:** 10.1371/journal.pone.0197734

**Published:** 2018-05-24

**Authors:** Christopher R. Ellis, Naomi L. Kruhlak, Marlene T. Kim, Edward G. Hawkins, Lidiya Stavitskaya

**Affiliations:** Center for Drug Evaluation and Research, United States Food and Drug Administration, Silver Spring, Maryland, United States of America; UMR-S1134, INSERM, Université Paris Diderot, INTS, FRANCE

## Abstract

Opioids represent a highly-abused and highly potent class of drugs that have become a significant threat to public safety. Often there are little to no pharmacological and toxicological data available for new, illicitly used and abused opioids, and this has resulted in a growing number of serious adverse events, including death. The large influx of new synthetic opioids permeating the street-drug market, including fentanyl and fentanyl analogs, has generated the need for a fast and effective method to evaluate the risk a substance poses to public safety. In response, the US FDA’s Center for Drug Evaluation and Research (CDER) has developed a rapidly-deployable, multi-pronged computational approach to assess a drug’s risk to public health. A key component of this approach is a molecular docking model to predict the binding affinity of biologically uncharacterized fentanyl analogs to the mu opioid receptor. The model was validated by correlating the docking scores of structurally diverse opioids with experimentally determined binding affinities. Fentanyl derivatives with sub-nanomolar binding affinity at the mu receptor (e.g. carfentanil and lofentanil) have significantly lower binding scores, while less potent fentanyl derivatives have increased binding scores. The strong correlation between the binding scores and the experimental binding affinities suggests that this approach can be used to accurately predict the binding strength of newly identified fentanyl analogs at the mu receptor in the absence of *in vitro* data and may assist in the temporary scheduling of those substances that pose a risk to public safety.

## Introduction

The severe social and economic impact of the opioid health crisis in the United States cannot be overstated. While opioids have provided an invaluable treatment option for managing pain, their addictive nature has contributed to the current crisis that has devastated countless communities[1, 2].

There is no single panacea to end the epidemic, and a multifaceted, widespread effort is underway to combat the crisis. Increased physician and patient education and tighter limits on opioid advertising lead an effort to reduce opioid over-prescribing [3]. Drug developers are designing abuse-deterrent formulations and developing safer ‘atypical/biased’ opioids that aim to eliminate rewarding effects and reduce the risk of respiratory depression, the primary cause of opioid overdose death [4–6]. While these atypical/biased opioids may provide safer alternatives to traditional opioids, they may not reduce the addictiveness of opioids and new pain treatment medications that target novel enzymes or receptors are needed [3]. Furthermore, sustained addiction treatment is required for recovering addicts as many years of treatment are often necessary for full recovery [6, 7].

Heroin and synthetic opioids are primarily responsible for the dramatic increase in the number of drug overdose deaths, despite oxycodone and hydrocodone being the most commonly abused [2, 8]. In particular, opioid-related overdose deaths due to synthetic opioids such as fentanyl and its analogs increased 72.2% from 2014 to 2015 and the trend is not slowing down or decreasing [1, 9]. The straightforward synthesis, low cost, and high potency of fentanyl have contributed to the influx of fentanyl analogs into the street-drug market as indicated by the DEA 2016 Annual Emerging Threat Report, which identified over 80% of emerging opioids as fentanyl analogs. The variability in potency among fentanyl analogs also presents a substantial risk to public health as some derivatives, e.g. carfentanil, are estimated to be about 10,000 times more potent than morphine [10, 11].

**Fig 1:**
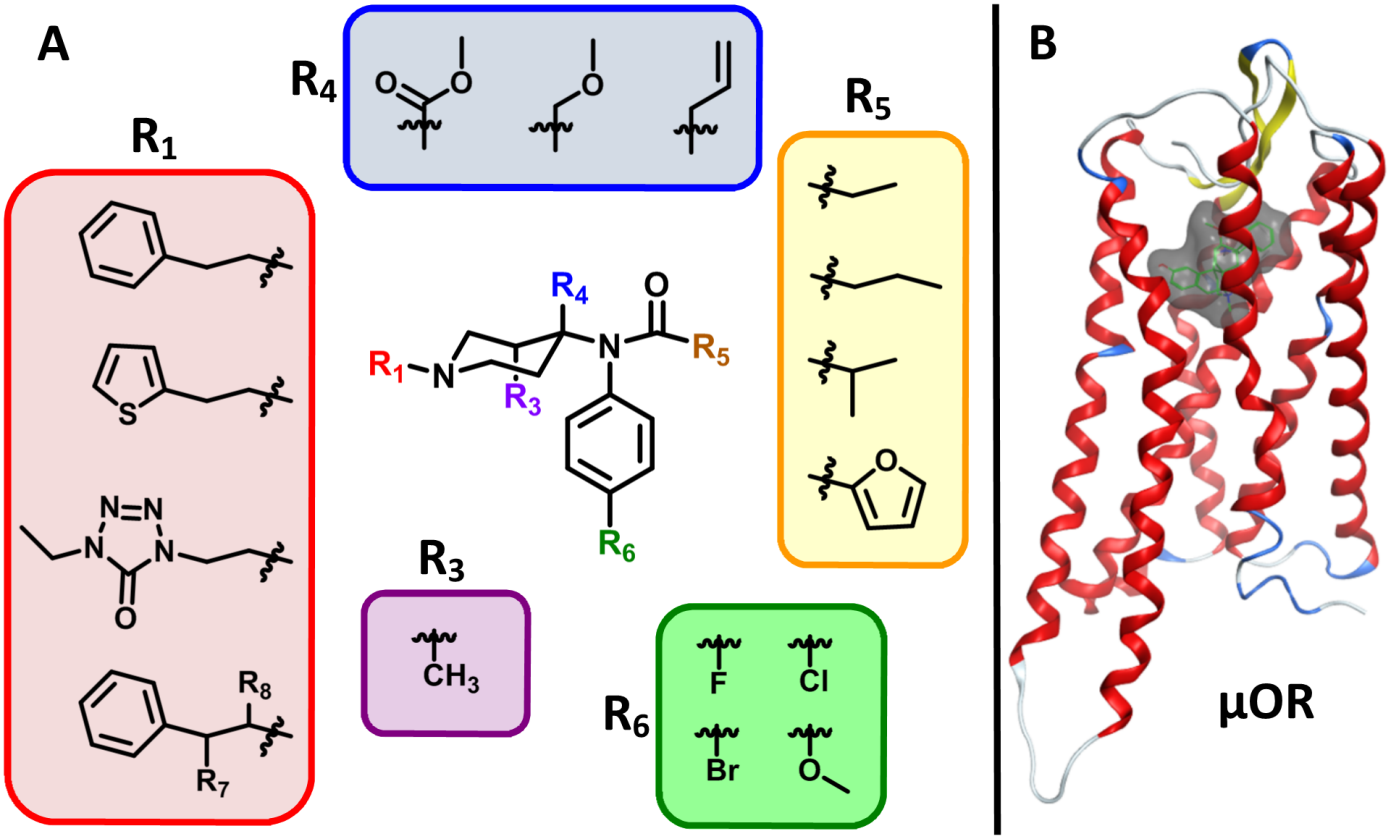
Fentanyl derivatization and the μOR. (A) Commonly-modified positions around the 4-anilidopiperidine core of fentanyl. (B) The seven-helix transmembrane domain of the μOR in complex with agonist BU72; the binding site is represented by the transparent surface.

Fentanyl is a mu opioid receptor (μOR) agonist that causes analgesic and euphoric effects. Given the high potency of fentanyl, it is commonly ‘cut’ into heroin, which has contributed to the increase in heroin overdoses and deaths [2]. The fentanyl influx is exacerbated by the ease with which simple modifications to the core fentanyl structure (4-anilidopiperidine, Fig 1A) can yield potent analogs. Specifically, modification of the central fentanyl piperidine ring (R_3_ and R_4_) has led to the development of powerful derivatives such as carfentanil and lofentanil [12]. In contrast, removal of the *N*-phenethyl (R_1_) group greatly reduces binding affinity at the μOR [13]. Importantly, many slight structural modifications do not alter the function and primary binding modes with the μOR creating a vast chemical space of fentanyl analogs with abuse potential. The Analogue Act (21 U.S.C. 813) was designed to assist in the prosecution of chemical analogs, but it has created a significant burden to demonstrate that two chemicals are ‘substantially similar’ and not intended for human consumption each time the drug is detected. This has led to significant challenges in the regulation of novel fentanyl analogs that evade control by national and international legislation.

To address the risk a substance poses to public health, the Drug Enforcement Administration (DEA) can assign the substance to a legislative “schedule” based on its psychological and/or physiological abuse potential. Substances with a high abuse potential and no accepted medical use are placed into Schedule I, while substances with an accepted medical use and varying degrees of abuse potential are placed into Schedules II-V. Schedule II narcotics include opioids approved for pain management, such as morphine, fentanyl, and codeine, while Schedule I narcotics include heroin. However, there is often no scientific knowledge regarding newly-identified fentanyl analogs and a complete eight-factor analysis to support a scheduling action can take up to two years. The eight-factor analysis includes experimental investigations of a new drug’s pharmacology, mechanism of action, abuse potential, and public health risks. In response to this need, the US FDA’s Center for Drug Evaluation and Research (CDER) has developed a molecular docking model that harnesses the concepts of virtual screening and simulates the binding interactions of biologically uncharacterized fentanyl analogs at the μOR to predict binding affinity. The model is designed to be used in combination with the complementary approaches of structural similarity assessment [14], target identification [15, 16], and functionality prediction [17, 18] to provide a comprehensive assessment of abuse potential to support temporary scheduling actions and provide medical professionals with risk assessment information in an emergency public health situation. This report describes the development, validation, and application of this molecular docking model to fentanyl analogs and other opioids, as part of a translatable approach with broader application to other classes of abused substances.

## Methods

Virtual screening—a computational drug identification and optimization protocol—has enjoyed success in the pharmaceutical industry for structure-based drug design applications [19]. The ever-increasing number of pharmacological targets and the tremendous magnitude of potential chemical space has created the need for rapid, low-cost predictions of activity over expensive, experimental measurement using traditional techniques [20, 21]. Molecular docking, a type of virtual screening, places compounds or ligands into potential binding poses within the binding site of a biological target and assesses binding affinity with a scoring function. The scoring function evaluates the non-covalent interactions between the ligand and target to provide a binding energy (score) which can be used to rank compounds with increasing binding affinity [22–24]. Initially, the molecular details of the docking simulations, e.g. docking poses and scoring distributions, are presented for a series of fentanyl analogs. These compounds have high structural similarity but significantly different binding affinities that range from sub-nM to μM. The model is further validated by docking and scoring a structurally diverse set of twenty-three opioids. The strong correlation between the docking score and binding affinities allows for the classification of the opioids into three binding concentration regimes based on the docking score.

A series of 23 opioids were docked with the μOR crystal structure (Fig 1B, PDBID: 5C1M [25]). The set of opioids contains eight fentanyl analogs (*N*-methyl fentanyl, *N*-methyl carfentanil, fentanyl, alfentanil, sufentanil, carfentanil, lofentanil, and R30490), seven fentanyl congeners ((±)-tramadol, meperidine, propoxyphene, diphenoxylate, and (±)-methadone) and eight morphine derivatives (codeine, (+)-pentazocine, oxycodone, nalbuphine, morphine, oxymorphone, hydromorphone, and buprenorphine). The μOR was crystallized in the active state with the agonist BU72 occupying the binding site. The crystal structure was prepared using the ‘QuickPrep’ function within Molecular Operating Environment (MOE) [26]. ‘QuickPrep’ sets the protonation states of all titratable residues within the protein structure and performs an energy minimization to remove any steric clashes. All water molecules in the crystal structure were maintained in the structure preparation and included during docking and pose evaluation. Each opioid was docked using the Triangle Matcher placement algorithm, and refined using the induced fit protocol. A cation pharmacophore was placed at the positively charged amine of BU72, a key interaction critical for amine-based opioid binding [27]. The pharmacophore is used as a suggestion for initial placement; however, the binding pocket and drug are relaxed (induced fit) upon ligand docking.

The protein and small molecules were modeled with the Amber force field in combination with the Extended Hückel Theory parameterization for small molecules [28–30]. The docked drugs are scored with the GBVI/WSA scoring function [31]. All drugs were docked in the protonated form which imparts chirality at the positively charged amine. All fentanyl and phenylpiperidine derivatives placed the hydrogen in the axial position with the R-group in the equatorial position. The morphine derivatives were docked in both protomeric states and the lowest average docking score was used. Given the stochastic nature of the docking algorithm, there is some variability in the best docking pose and score from each simulation. Therefore, each opioid was docked and scored in ten independent simulations and the average of the lowest energy pose is used in the following analysis. The entire procedure was performed in triplicate to ensure reproducibility. The average docking score (ADS) of the best pose from the three sets of simulations are within 0.2 kcal/mol for each drug (Supporting Information, Fig 1).

The method was further evaluated by docking a series of 50 presumed non-binding fentanyl decoys generated by the Directory of Useful Decoys, Enhanced (DUD-E) [32, 33]. DUD-E was used to generate decoy structures that are physically similar to fentanyl but vary topologically. The difference in molecular shape should reduce the binding affinity. The 50 decoys contained at least one positively charged amine and were docked and refined using the previously described docking protocol. Finally, the structural similarity of fentanyl with respect to the decoys was assessed using a combination of MACCS keys (166-bit) from MOE and the Tanimoto similarity index [14]. The Tanimoto similarity index, or Tanimoto coefficient (T_c_), ranges from 0–1. Compounds with a high degree of structural similarity have a T_c_ near one, while structurally divergent compounds have a score near zero.

## Results and discussion

### Molecular docking and scoring

**Fig 2:**
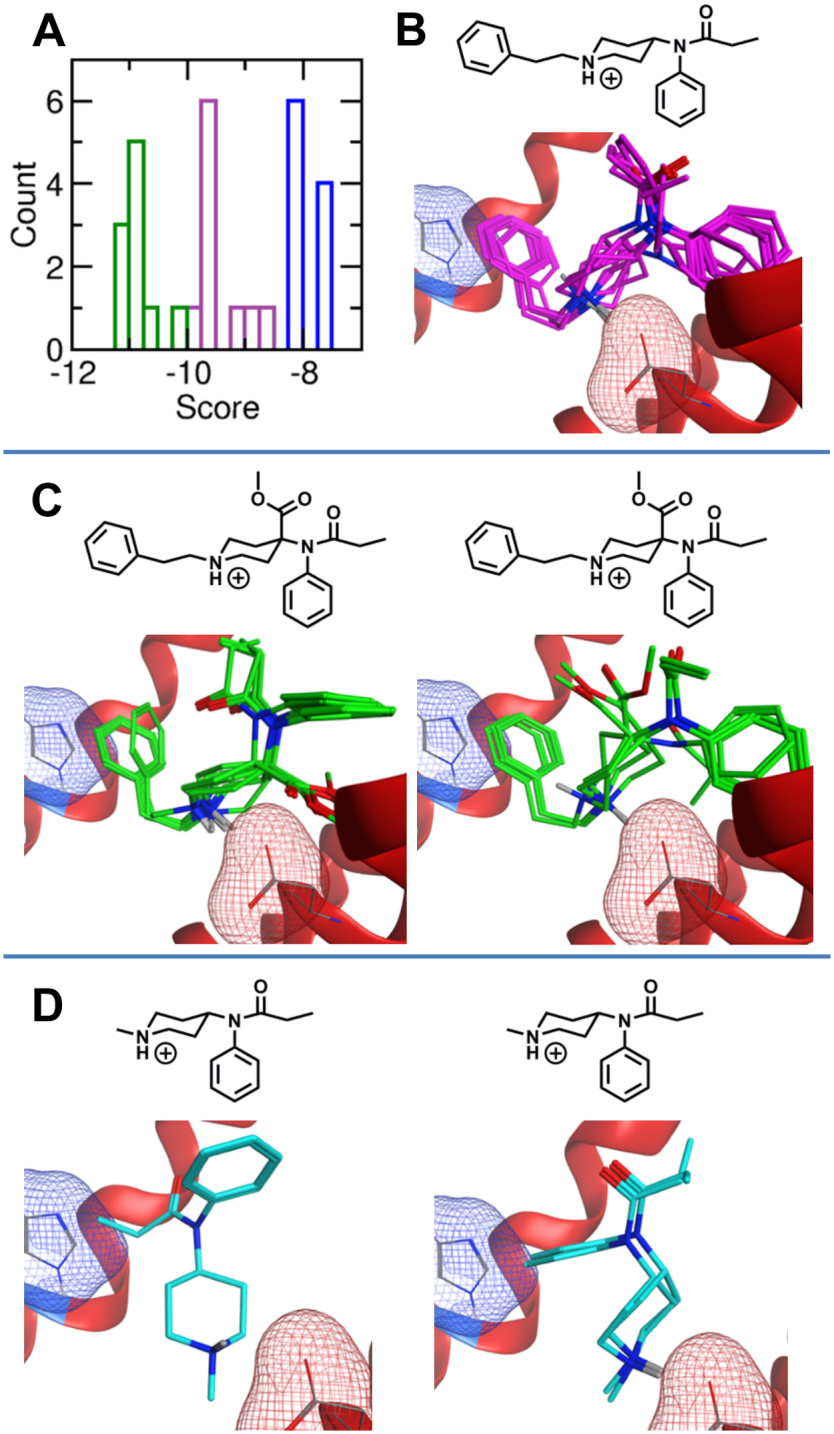
Molecular docking poses and scores. (A) Docking score distribution from the 10 docking simulations of fentanyl (purple), carfentanil (green) and *N*-methyl fentanyl (blue). (B) Superimposition of the ten lowest energy fentanyl poses in the μOR binding pocket. (C) The left and right panels present the primary (7) and secondary (3) carfentanil poses from the 10 docking simulations, respectively. (D) The left and right panels present the primary (6) and secondary poses (4) of *N*-methyl fentanyl from the ten docking simulations, respectively. The six primary *N*-methyl fentanyl poses are virtually identical. In panels B-D, the negatively charged sidechain of Asp147 that forms the salt bridge with the positively charged amine of each opioid is highlighted by the red mesh surface, and the aromatic sidechain of His297 is represented by the blue mesh surface.

The molecular docking and scoring procedure distinguishes between the binding affinities of fentanyl, carfentanil, and *N*-methyl fentanyl despite the similar structures and relates the increased binding affinity to the underlying molecular interactions. Fentanyl (K_i_ = 1.35 nM) was independently docked to the μOR ten times and received an ADS of -9.4 kcal/mol. The lowest energy (i.e. best) fentanyl poses are nearly identical from each simulation. However, the slight variation in pose and active site conformation gives rise to fluctuations in the score from each independent simulation (Fig 2A and 2B). Each minimum energy pose maintains the salt bridge between the positively charged amine and the negatively charged aspartic acid (Asp147, red mesh surface Fig 2B–2D) that is critical for amine-based opioid binding [27]. Additionally, all poses contain aromatic stacking between the *N*-linked phenethyl (R_1_) and a histidine (His297, blue mesh surface Fig 2B–2D) residue within the binding pocket. Importantly, the aromatic stacking interaction was identified by the docking procedure and was not suggested by the pharmacophore.

Carfentanil contains a methyl ester at the 4-position of the fentanyl piperidine ring (Fig 1A, R_4_). Consistent with carfentanil’s increased binding affinity at the μOR (K_i_ = 0.22 nM), the carfentanil ADS is -10.8 kcal/mol, significantly lower than fentanyl. Similar to fentanyl, the lowest energy carfentanil poses maintain the salt bridge and aromatic stacking interactions within the binding pocket. Seven of the top 10 poses contain nearly identical placement, while 3 poses vary (Fig 2C).

Chemical modification at the fentanyl *N*-phenethyl moiety (R_1_) can also impact the binding affinity of fentanyl derivatives at the μOR. Strikingly, the *N*-benzyl analog of fentanyl only differs by the removal of a single carbon but greatly reduces the activity (ED_50_ = 10–45 mg/kg) compared to that of fentanyl (ED_50_ = 0.01 mg/kg)[34]. In contrast, replacement of the aromatic phenyl with other aromatic groups including thiophene and tetrazole, e.g. sufentanil and alfentanil, maintain strong binding with the μOR, while removal of the *N*-phenethyl group significantly reduces binding affinity at the μOR [13]. Indeed, *N*-methyl fentanyl is inactive at 100 mg/kg in mice [34]. Furthermore, the binding affinity of *N*-methyl fentanyl and *N*-methyl carfentanil are 15,000 and 1,750 times weaker than fentanyl and carfentanil at the guinea pig brain μOR, respectively [13].

Consistent with these experimental observations, the ADS of *N*-methyl fentanyl is significantly larger than the ADS of fentanyl. Although *N*-methyl fentanyl does contain an aromatic group, the compound is not large enough to maintain the salt bridge while simultaneously stacking with the μOR histidine. The lack of aromatic stacking increases the ADS of *N*-methylfentanyl to -7.9 kcal/mol. The distribution of *N*-methyl fentanyl scores is very narrow and has a small standard deviation. Interestingly, despite the small fluctuations in score, *N*-methyl fentanyl does not adopt a unique binding pose. *N*-methyl fentanyl adopts a primary conformation in six lowest energy structures and a secondary binding pose in the remaining four configurations (Fig 2D).

### Classifying opioid binding affinities with docking scores

The docking procedure was repeated for 23 opioids (Table 1). The 23 opioids (21 unique compounds and two stereoisomers) were selected to provide structural diversity across different classes of opioids. Ideally, the experimental binding data should be derived from the same source and measurements taken from human opioid receptors. However, experimental data derived from human μOR are limited and reported binding affinities from different experiments range dramatically depending on numerous experimental conditions including the choice of radioligand, tissue source, and species [35]. Perhaps the most striking reported binding affinity range is for fentanyl K_i_ measurements, ranging from 0.007 to 214 nM [36, 37].

**Table 1:** Experimentally determined and predicted binding affinity ranges of the 23 opioids docked at the μOR. The 8 fentanyl derivatives and 7 fentanyl congeners are listed first and ordered by increasing binding affinity, and the 8 morphine derivatives are listed last. The green labels indicate that the model correctly predicted the binding concentration regime, yellow indicates that the model predicted the compound to bind stronger than the measured K_i_, and red indicates that the model predicted the compound to bind less strongly than the measured K_i_.

Drug	Human K_i_ (nM) [35]	Marmoset K_i_ (nM) [38]	Guinea Pig K_i_ (nM) [13]	Average Docking Score (ADS)	Standard Deviation in Score	Predicted Conc. Regime
*N*-methyl fentanyl*			18000 ± 3000	-7.89	0.21	> 100 nM
(1R,2R)Tramadol	12,500			-7.85	0.06	> 100 nM
(1S,2S) Tramadol	12,500			-7.97	0.20	> 100 nM
Meperidine	450			-7.77	0.07	> 100 nM
Propoxyphene	120			-9.56	0.08	0–100 nM
*N*-Methyl carfentanil*			42 ± 6	-8.53	0.07	0–100 nM
Diphenoxylate	12.4			-10.1	0.47	Sub nM
Alfentanil	7.39	14.4 ± 4.2		-10.5	0.56	Sub nM
R-Methadone	3.38			-8.69	0.12	0–100 nM
S-Methadone	3.38			-8.63	0.07	0–100 nM
Fentanyl	1.35	1.32 ± 0.35	1.2 ± 0.2	-9.43	0.38	0–100 nM
Sufentanil	0.138	0.24 ± 0.05		-9.89	0.23	Sub nM
Carfentanil^		0.22 ± 0.08	0.024 ± 0.004	-10.8	0.30	Sub nM
Lofentanil^		0.055 ± 0.006	0.023 ± 0.004	-10.8	0.50	Sub nM
R30490*			0.09 ± 0.01	-10.3	0.31	Sub nM
Codeine	734			-8.23	0.16	> 100 nM
(+)-Pentazocine	118			-7.77	0.12	> 100 nM
Oxycodone	25.9			-8.78	0.27	0–100 nM
Nalbuphine	2.12			-8.47	0.29	0–100 nM
Morphine	1.14			-7.76	0.14	> 100 nM
Oxymorphone	0.406			-8.40	0.18	0–100 nM
Hydromorphone	0.365			-7.94	0.26	> 100 nM
Buprenorphine	0.216			-9.76	0.48	0–100 nM

*Experimental data from guinea pig whole brain membranes.

^Experimental data from marmoset brain homogenates.

In order to maximize the number of docked and scored compounds, the experimentally determined binding affinities were taken from three sources [13, 35, 38]. The binding affinities of 18 of the 23 compounds were measured in competitive binding assays that displaced (^3^H)-DAMGO from recombinant human μOR [35]. In a similar competitive binding assay, the binding affinities of carfentanil and lofentanil were measured from marmoset brain homogenates [38]. Finally, binding affinities of *N*-methyl fentanyl, *N*-methyl carfentanil and R30490 were measured from guinea pig whole membranes [13]. Despite differences in the animal source, the measured binding affinity of fentanyl was between 1.2 and 1.4 nM across the three experiments. Additionally, the measured K_i_ of alfentanil and sufentanil were approximately two times weaker in the marmoset brain homogenates compared to the recombinant human receptors. Moreover, carfentanil and lofentanil are known to have stronger interactions with the μOR, while the *N*-methyl derivatives have decreased binding affinity.

#### Fentanyl analogs

**Fig 3:**
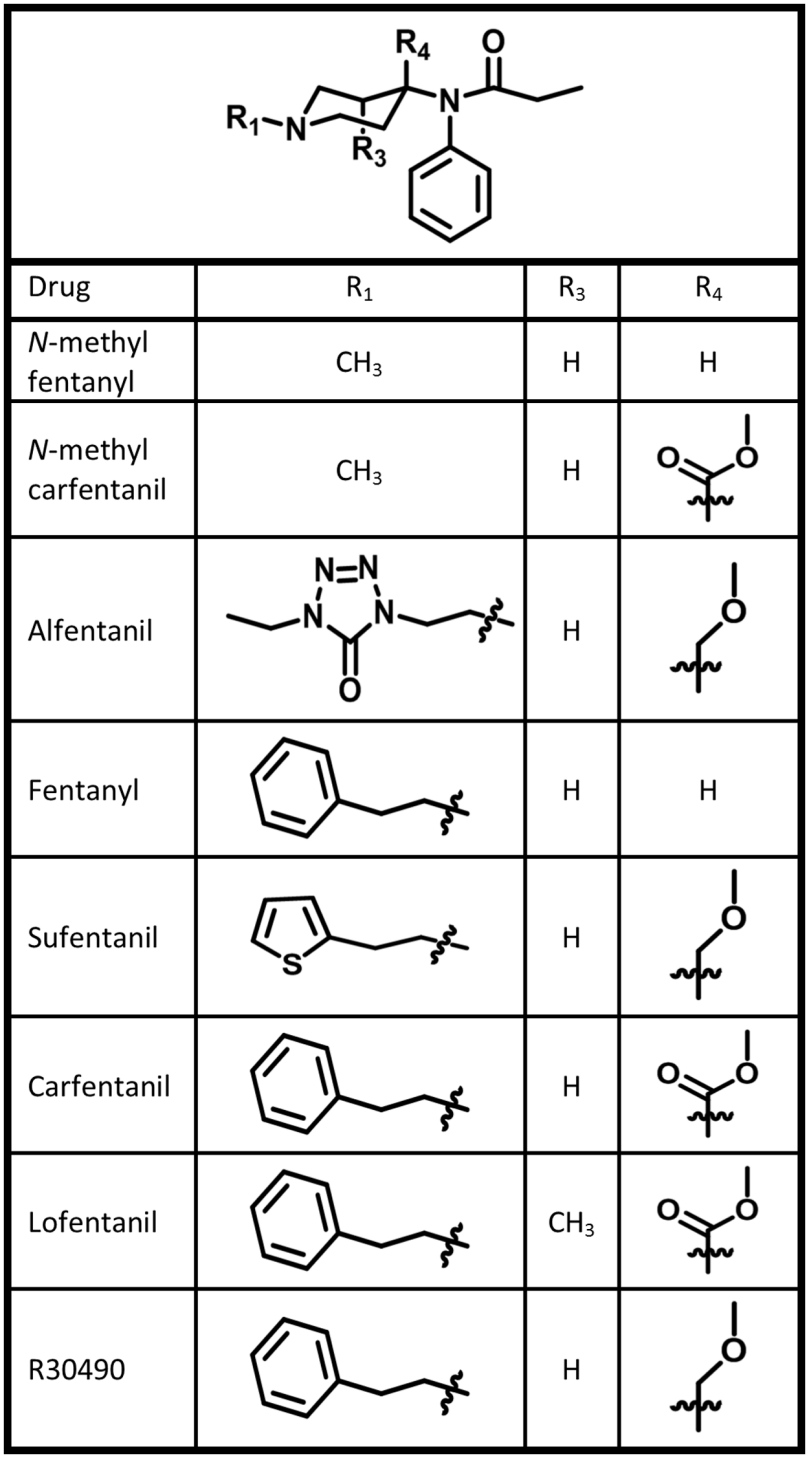
Fentanyl analog structures. Summary of the fentanyl analog chemical features assessed by molecular docking.

The series of docked and scored fentanyl analogs range from sub-nM to μM binding affinities and explicitly investigate the effect of chemical modifications at critical locations within the fentanyl scaffold. Specifically, the impact of (1) inclusion of a polar group at the 4-position of the piperidine ring (carfentanil, lofentanil, R30490), (2) aromatic substitution on the *N*-linked substituent (sufentanil, alfentanil), (3) methyl substitution at the third position of the piperidine ring (lofentanil), and (4) removal of the aromatic ring from the *N*-linked substituent (*N*-methyl fentanyl, *N*-methyl carfentanil) is tested (Fig 3).

**Fig 4:**
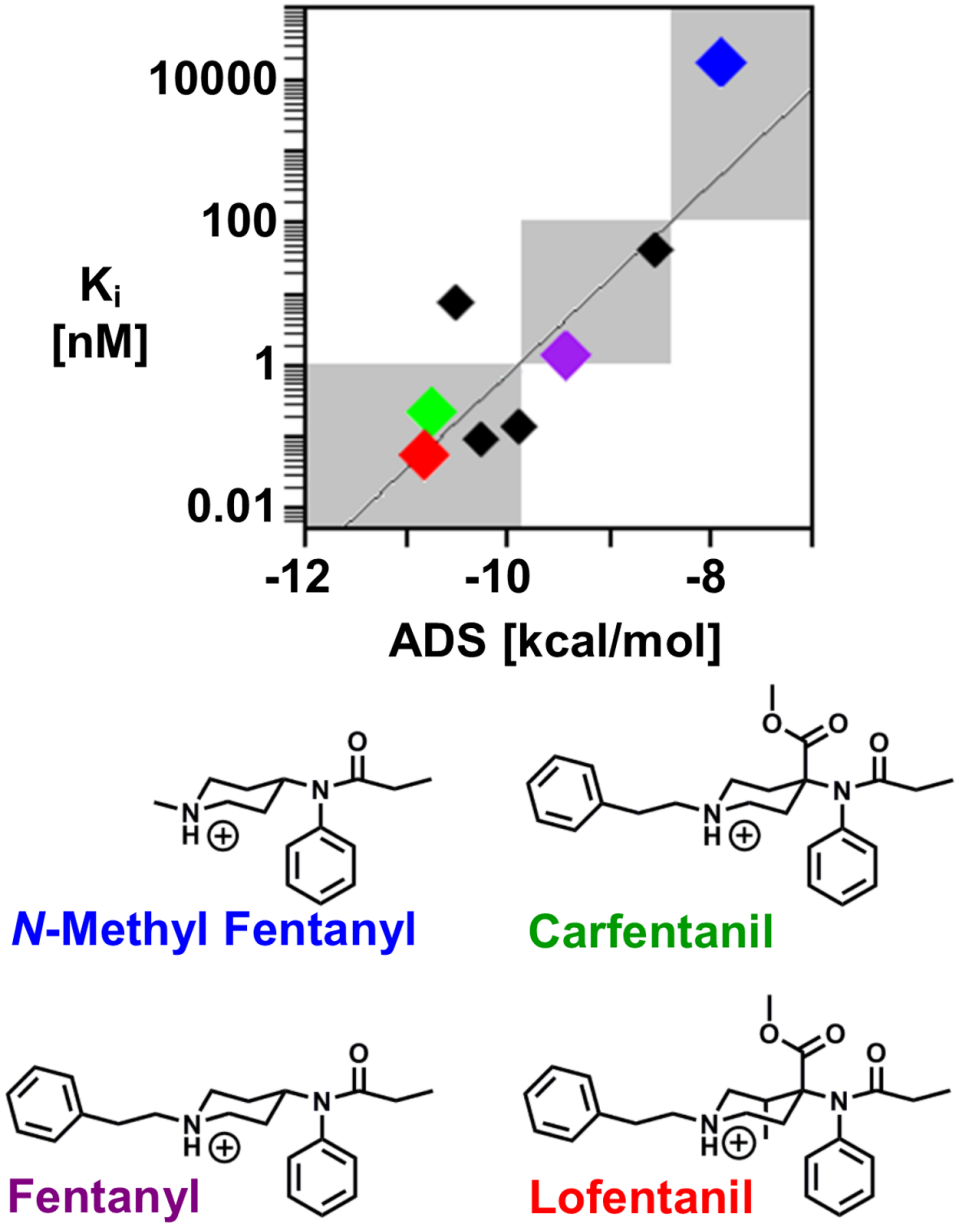
Fentanyl analog binding prediction and classification. Scatterplot of the experimentally determined binding affinity, K_i_, with the ADS from the molecular docking procedure of the 8 fentanyl analogs (shown as diamonds). The grey, shaded regions display the sub-nM, 1–100 nM, and greater than 100 nM binding concentration regimes used to classify the predicted binding strength of new drugs. *N*-methyl fentanyl (blue), fentanyl (purple), carfentanil (green) and lofentanil (red) are presented with colored diamonds to demonstrate the method’s ability to separate fentanyl analogs into the proper binding concentration regimes. The remaining black diamonds represent the 4 other fentanyl analogs that were docked and scored.

The fentanyl analogs include 4 strong binders (K_i_ < 1 nM), 3 moderate binders (1 < K_i_ < 100 nM), and 1 weak binder (K_i_ > 100 nM). The strong correlation between the experimentally determined binding affinity and calculated ADS of the fentanyl analogs (r = 0.86) allows for separation into the three predicted binding concentration ranges, or regimes (Fig 4). Compounds that receive an ADS of less than -9.9 are predicted strong binders, compounds that receive an ADS of between -9.8 and -8.4 are predicted moderate binders, and compounds that receive an ADS of greater than -8.4 are predicted weak binders. The ADS correctly assigns the binding concentration regime of all but one fentanyl derivative, alfentanil. The measured binding affinity of alfentanil is 7.4 nM; however, the docking procedure predicts sub-nM binding affinity (ADS = -10.5). The low ADS is likely due to docking alfentanil in the positively charged state. Unlike most fentanyl derivatives, the pKa of alfentanil is reduced and it is predominately neutral at physiological pH [39].

Carfentanil (ADS = -10.8, green), fentanyl (ADS = -9.4, purple), and *N*-methyl fentanyl (ADS = -7.8, blue) bind in the sub nM, 1–100 nM, and greater than 100 nM binding concentration regimes, respectively. Carfentanil, an opioid only intended for animal use because its potency makes it inappropriate for humans [12], is almost structurally identical to lofentanil (ADS = -10.8, red). The methyl substitution at the 3-position gives rise to a marked increase in the lofentanil binding affinity [38], however, both carfentanil and lofentanil receive nearly identical binding scores from the docking procedure (carfentanil ADS = -10.75, lofentanil ADS = -10.82). The scoring function is likely unable to distinguish between these two compounds because the additional methyl within the piperidine ring does not significantly change the molecule’s shape or impact the binding poses.

Importantly, the predictions distinguish between key structural features of fentanyl analogs that either increase or decrease the binding affinity. Specifically, compounds with chemical modifications at the 4- position within the piperidine ring increase binding affinity, removal of the *N*-phenethyl group significantly diminishes binding affinity, and exchange for a different aromatic group at the *N*-phenethyl group does not significantly alter the binding score. However, the slight modification of the additional methyl within the ring is too subtle to be picked up by the scoring function.

#### Fentanyl congeners

**Fig 5:**
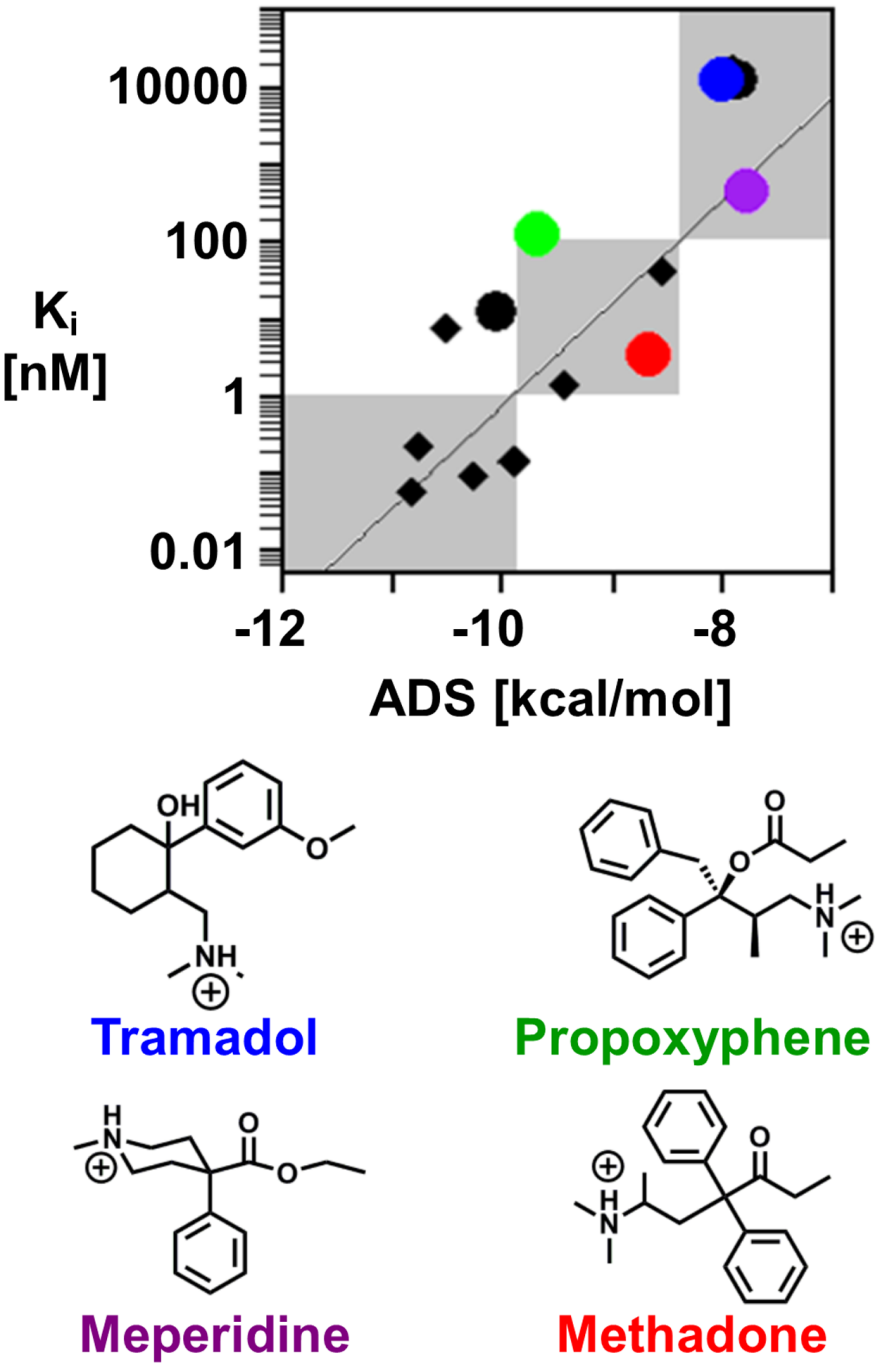
Fentanyl congener binding prediction and classification. Scatterplot of the experimentally determined binding affinity, K_i_, with the average docking score from the molecular docking procedure of the 7 fentanyl congeners (shown as circles). (+)-Tramadol (blue), meperidine (purple), propoxyphene (green) and methadone (red) are highlighted and demonstrate the method’s ability to predict the correct binding concentration regimes of fentanyl congeners. The remaining black circles represent the three fentanyl congeners that were docked and scored. The black diamonds represent the fentanyl analogs from Fig 4.

As an extension to the initial set of fentanyl analogs, 7 fentanyl congeners were docked and scored. The fentanyl congeners include the phenylpiperidine derivatives, diphenoxylate and meperidine, and the noncyclic-aliphatic amines, (±)-tramadol, (±)-methadone, and propoxyphene. Similar to the fentanyl analogs, the fentanyl congener ADS correlates with the measured binding affinities (r = 0.60) at the μOR and correctly assigns the binding concentration regime in 5 of the 7 compounds assessed (Fig 5). The 2 incorrectly assigned compounds (propoxyphene and diphenoxylate) are predicted to bind more strongly than the measured binding concentration regime. Both enantiomers of tramadol and methadone were docked and scored because the experimental binding affinities were measured using racemic mixtures and the drugs are typically administered clinically as racemic mixtures [35].

Meperidine (purple) and (±)-tramadol (blue) were correctly assigned as weak binders. This is perhaps not surprising considering that these compounds are structurally similar to *N*-methyl fentanyl. While these drugs do contain an aromatic moiety, the separation between the positively charged amine and aromatic ring is not large enough for the salt bridge and aromatic stacking interactions to occur simultaneously. Propoxyphene (green), was incorrectly assigned to the moderate binding regime and received an ADS of -9.6 kcal/mol. However, the experimentally determined binding affinity is 120 nM, very close to the cut-off between the weak and moderate binding regime. Furthermore, an overestimated binding prediction is preferred to an underestimation of the binding affinity in the context of protecting public health.

Methadone (red), commonly associated with detoxification of patients with opioid dependence, is administered as a racemic mixture of R-methadone and S-methadone. However, previously published binding studies indicate that R-methadone has a 10-fold higher affinity for the μOR and accounts for most of the pharmacological effect [40, 41]. Despite the difference in binding affinities, each methadone enantiomer would be classified as a moderate binder in the given concentration regimes. Binding measurements from the data set used in the current study were based on a racemic mixture of methadone [35]. Therefore, given only one measured K_i_ for the racemic mixture, the correlation plot uses K_i_ = 3.38 nM for both R- and S-methadone, both of which receive an ADS of -8.7 kcal/mol.

#### Fentanyl decoys

**Fig 6:**
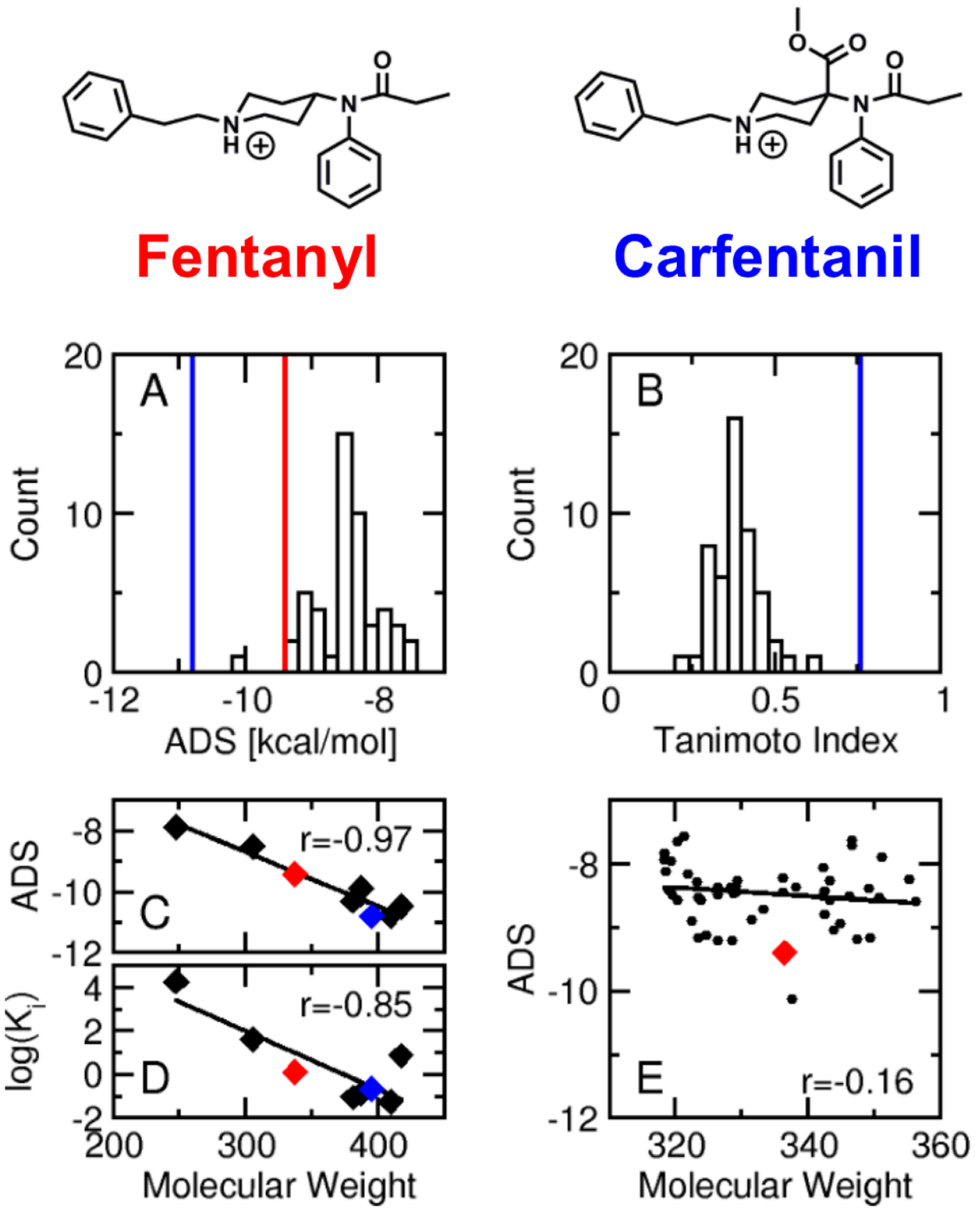
Decoy analysis. (A) Distribution of the decoy binding scores. The vertical red and blue lines indicate the docking score of fentanyl and carfentanil, respectively. (B) Distribution of the Tanimoto index of the fentanyl decoys with respect to fentanyl. The vertical blue line indicates the Tanimoto index of carfentanil (T_c_ = 0.76) with respect to fentanyl. (C) Correlation of the fentanyl derivative molecular weight with respect to the ADS. (D) Correlation of the fentanyl derivative molecular weight with respect to the experimentally determined binding affinity (K_i_). (E) Correlation of the fentanyl decoy molecular weight with respect to the ADS. In panels C-E, the red and blue diamonds indicate fentanyl and carfentanil, respectively.

The set of 50 fentanyl decoys, generated using the Directory of Useful Decoys, Enhanced (DUD-E) server [32, 33], was docked and scored at the μOR. The fentanyl decoys have similar molecular weight and chemical features to fentanyl, but vary in molecular shape. Decoys provide a comparison between an experimentally known binder and presumed non-binders providing a measure of how known ligands rank versus a background. The distribution of the fentanyl decoy ADS values (Fig 6A) demonstrates that 49 of the 50 decoys have a higher ADS than fentanyl (red vertical line), and the majority of the decoys receive an ADS of approximately 1 kcal/mol higher than fentanyl. The single decoy with a lower ADS than fentanyl (ADS = -10.1) contains two positively charged amines in close proximity to each other forming a dual salt bridge with the negatively charged aspartic acid driving the docking score lower (predicted stronger binding). All 50 decoys had substantially lower ADS values than carfentanil (vertical blue line, Fig 6A). Next, the distribution of the decoy Tanimoto coefficients (T_C_, Fig 6B) quantifies the structural similarity of the decoys with respect to fentanyl and demonstrates that the decoys are structurally dissimilar to fentanyl (average T_c_ = 0.39). In contrast, the fentanyl analogs sufentanil (T_c_ = 0.69) and carfentanil (T_c_ = 0.76, blue vertical line) have much higher similarity scores with respect to fentanyl. Finally, this model was developed to prospectively predict the binding affinity of newly identified drugs of abuse at the μOR, where some drugs may not bind. Accordingly, chemicals that are not known binders with a predicted binding regime of K_i_ >100 nM must be considered weak or non-binding.

Binding affinity predictions using molecular docking can be challenging due to inherent limitations with scoring functions. Larger molecules can form more interactions within the binding pocket leading to lower binding scores. Indeed, the ADS score of the fentanyl derivatives is strongly correlated to the molecular weight (r = -.97, Fig 6C). However, the measured binding affinity (K_i_) is also strongly correlated with the molecular weight (r = -0.85, Fig 6D). Alfentanil is the only chemical that does not fall on the trend line, and as previously discussed, alfentanil is unique in that it is predominately neutral at physiological pH which may explain the reduced binding affinity [39]. Removing alfentanil from the molecular weight versus binding affinity correlation increases the correlation coefficient to -0.97. The lack of correlation between the fentanyl decoy’s ADS values and molecular weight (r = - 0.16, Fig 6E) demonstrates that the model and scoring function are not simply predicting using the molecular weight. The fentanyl decoy molecular weights span about 40 Da and the decoys generally score about 1 kcal/mol higher than fentanyl. In contrast, R30490 only differs from fentanyl by a small ether group (R_4_, molecular weight = 45 Da) and decreases the ADS by 1 kcal/mol. In summary, the fentanyl decoy analysis demonstrates that the model ranks fentanyl as a stronger binder than compounds with similar chemical features but different molecular shapes, and the scoring function is not simply predicting based on the molecular weight. The model properly assesses chemical features that lead to increased or decreased binding affinity.

#### Application to designer drugs

**Fig 7:**
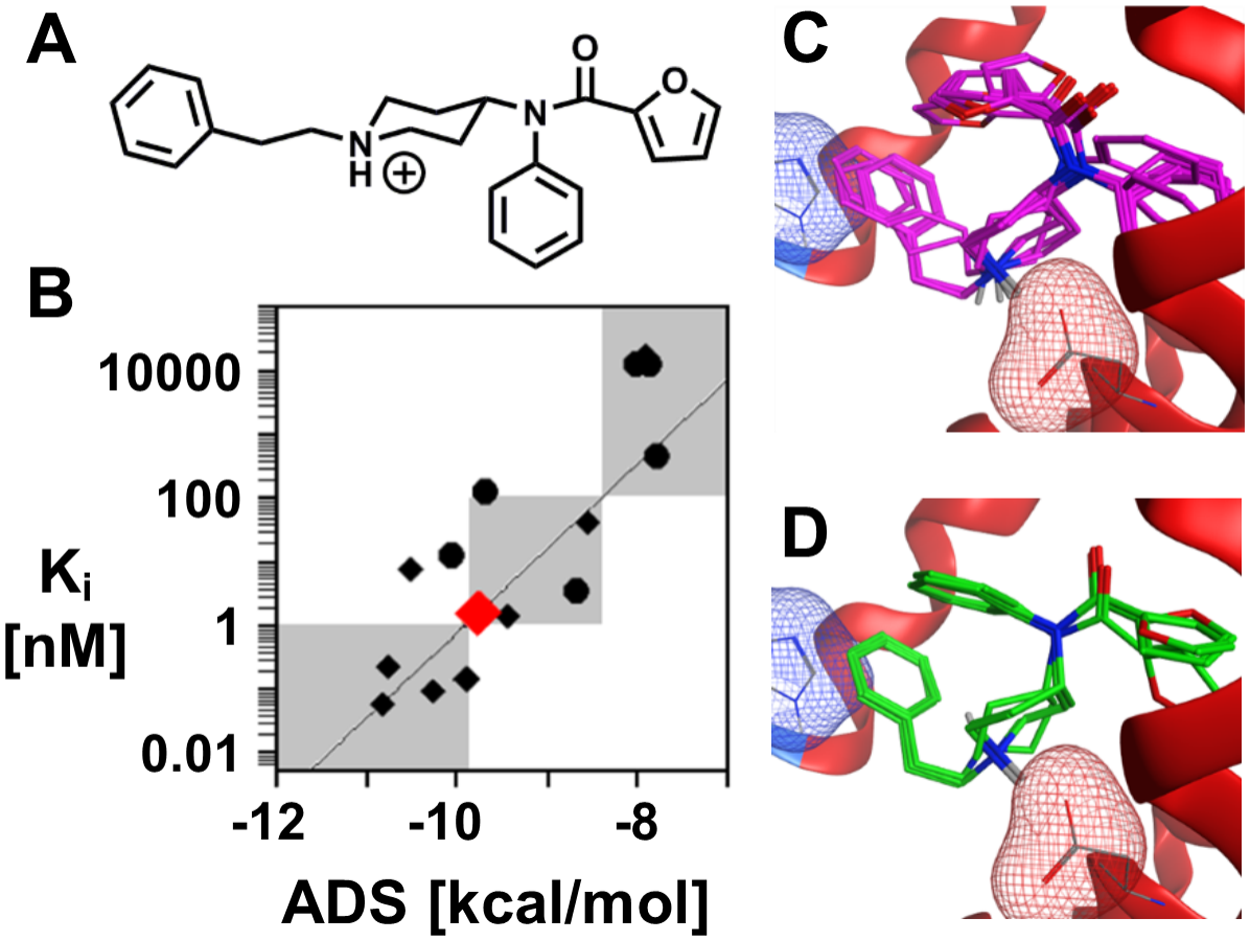
Furanylfentanyl case study. (A) Chemical structure of the designer opioid Fu-F. (B) Binding concentration prediction of Fu-F (red). (C-D) Primary and secondary binding poses from the 10 independent docking simulations of Fu-F. In panels C and D, the key salt bridge (Asp147) and aromatic stacking interactions (His297) with Fu-F are highlighted by the red and blue mesh surfaces, respectively.

Newly-identified fentanyl analogs generally lack any *in vitro* and/or *in vivo* data. To fill this data gap, the docking and scoring methodology can serve as a prediction protocol for assessing the binding affinity of newly discovered fentanyl analogs at the μOR. For example, furanylfentanyl (Fu-F, Fig 7A), a designer fentanyl analog, has been confirmed in several drug overdoses [42–44], and the DEA has recently issued a final order to temporarily schedule Fu-F as a Schedule I narcotic to protect public safety [45].

Docking and scoring Fu-F with the μOR provides the predicted binding concentration regime (Fig 7B), as well as insight into the molecular details of the binding interactions. Fu-F shares many structural features with fentanyl including the phenethyl side chain (R_1_) and hydrogens at the 3- and 4- positions on the piperidine ring (R_3_ and R_4_). Given the common structural features, the Fu-F ADS (-9.6 kcal/mol, Fig 7B) is also similar to the fentanyl ADS (-9.4 kcal/mol). The lowest energy poses of Fu-F are analogous to fentanyl, the phenethyl (R_1_) stacks with histidine within the binding pocket and the benzene ring is directed toward the interior of the protein (Fig 7C). Fu-F contains a furan ring adjacent to the carbonyl, while fentanyl contains an aliphatic chain. Interestingly, the addition of the second aromatic ring allows for an alternative pose to be populated, which places the furan ring into the pocket that is occupied by the benzene ring in the primary Fu-F pose and all fentanyl poses (Fig 7D). All Fu-F poses maintain the salt bridge between the positively charged amine with the negatively charged aspartic acid and aromatic stacking within the binding pocket.

A multi-pronged approach that utilizes molecular docking, structural similarity assessment with previously scheduled substances, and biological target identification can provide a wealth of information regarding a new drug in the absence of *in vitro* and/or *in vivo* data. The atomistic view into the binding pocket of receptors in complex with new drugs provides insight into the molecular features that govern binding strength. As the number of fentanyl derivatives ‘cut’ into street drugs increases, *in silico* scientific assessment of new substances of abuse may provide rapid support for temporary scheduling of the new substances.

#### Limitations

**Fig 8:**
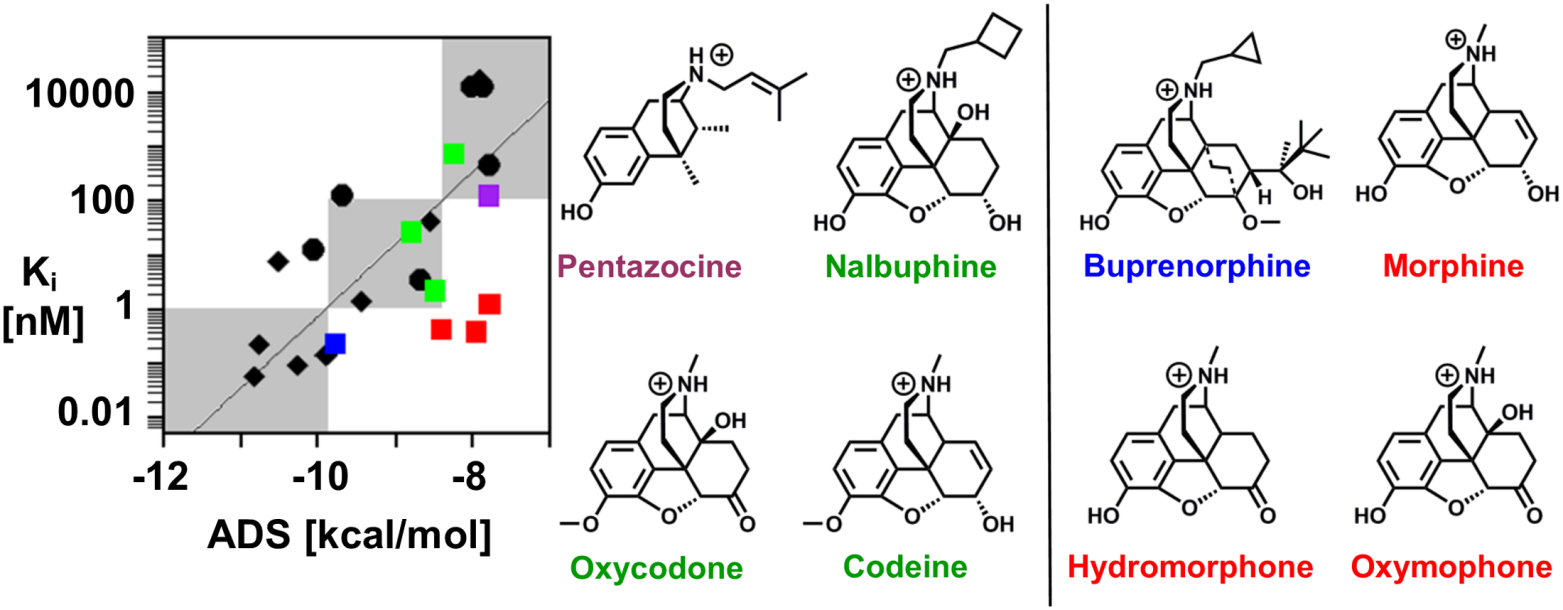
Morphine analogs: Binding prediction and classification. Scatterplot of the experimentally determined binding affinity, K_i_, with the ADS from the molecular docking procedure of the 8 morphine analogs (shown as squares). Pentazocine (purple) and buprenorphine (blue) are the most structurally dissimilar to morphine. The remaining 6 drugs are structurally similar to morphine; 3 are predicted correctly (green) and 3 are predicted incorrectly (red). The black diamonds and circles represent the fentanyl analogs and fentanyl congeners from Fig 5. For clarity, correctly and incorrectly predicted morphine analogs are on the left and right side of the vertical black line, respectively.

Eight morphine analogs were docked and scored at the μOR (Fig 8). In contrast to the fentanyl derivatives and fentanyl congeners, the ADS and experimental binding affinity is not strongly correlated (r = 0.32), and the docking and scoring procedure was unable to identify the correct binding concentration regimes of the morphine derivatives with high accuracy. Of the 8 derivatives, only 4 were properly predicted (2 weak binders, 2 moderate binders). Similar to carfentanil and lofentanil, the morphine analogs are too structurally similar for the scoring function to distinguish between the analogs. With the exception of pentazocine (purple), buprenorphine (blue), and nalbuphine which contain bulky hydrophobic groups at the positively charged amine, the remaining analogs are very structurally similar in the core structure and contain a methyl amine.

The docking procedure correctly predicts the binding concentration regime of codeine, oxycodone, and nalbuphine (green, left of the vertical black line). The ADS of these 3 compounds spans -8.8 to -8.2 kcal/mol, close to the separation of binding concentration regimes at -8.4 kcal/mol. However, the docking procedure incorrectly predicts the binding concentration regime of morphine, oxymorphone and hydromorphone (red, right of the vertical black line) and the ADS spans -8.4 to -7.8 kcal/mol. The molecular docking procedure predicts very similar scores for both the correctly and incorrectly predicted morphine analogs (-7.8 to -8.8 kcal/mol). The correct predictions for 3 out of the 6 structurally similar morphine analogs are driven by the experimentally determined binding affinity and not the uniqueness in structure. However, buprenorphine (Ki = 0.216 nM), which is structurally dissimilar, received a lower ADS and was nearly predicted to be a sub-nM binder. This highlights the limitation of the current model. The scoring function readily identifies and relates large structural changes, e.g. removal of an aromatic ring or the introduction of a large polar group, to the binding affinity. However, the scoring function is not sensitive enough to detect subtle structural changes, such as the addition of a methyl or the replacement of a hydroxyl group with a carbonyl. Despite the limitation observed with morphine analogs, the docking model provides an accurate assessment of μOR binding of fentanyl analogs, which constitute the most significant threat to public health from the opioid class. Indeed, according to the DEA 2016 Annual Emerging Threat Report, no newly-identified opioids in 2016 were morphine-based, suggesting that this limitation is unlikely to diminish the model’s practical utility.

## Conclusions

The rapid influx of fentanyl analogs on the street market has created the need for a rapid evaluation method to assess a new drug’s risk to public safety. Slight structural modifications to the fentanyl core can lead to a wealth of novel structures. As a result, pursuing enforcement actions against synthetic drug distribution for abuse purposes is challenging. When there is a need for temporary (or emergency) scheduling of a substance, a computational structure-based assessment that utilizes the molecular docking model described in this study may be used to provide early scientific support to determine if a substance may be an imminent hazard to public safety. A structure-based assessment strategy may also include structural similarity analysis [14], biological target identification [15, 16], and functionality prediction [17, 18], where the molecular docking model complements the biological target prediction to provide an estimated binding affinity.

The presented molecular docking and scoring procedure accurately predicts the binding concentration of fentanyl analogs and fentanyl congeners at the μOR. The molecular docking and scoring procedure is general and readily extends to any drug class and any receptor with an available crystal structure. However, it is critical that each drug class is assessed at the appropriate receptor to confirm the scoring function’s ability to determine how chemical modifications impact binding interactions and strength. Given the large influx of new fentanyl analogs ‘cut’ into street-drugs, a computational structure-based assessment strategy provides a rapid and low-cost evaluation to assess the risk a new drug poses to public health. This assessment could help guide the DEA and hospital workers when encountering these dangerous substances, both by aiding in scheduling decisions and potentially informing patient treatment.

## Supporting information

S1 Fig Average docking score. The docking procedure (ten independent simulations and averaging the best pose) was repeated three times for the 24 opioids. This plot shows the average docking score for run 1 (black), run 2 (red), and run 3 (blue).
(ZIP)
