# Supplementary material for: Predicting opioid receptor binding affinity of pharmacologically unclassified designer substances using molecular docking
Source: PLoS One. 2018 May 24;13(5):e0197734. doi: 10.1371/journal.pone.0197734 (PMC5967713; doi:10.1371/journal.pone.0197734)
Supplement: S1 Fig — The docking procedure (ten independent simulations and averaging the best pose) was repeated three times for the 24 opioids. This plot shows the average docking score for run 1 (black), run 2 (red), and run 3 (blue). (ZIP) [file pone.0197734.s001.zip › SI/SupportingInformation-OpioidDocking.pdf]

Supporting Information: Predicting the binding affinity of pharmacologically unclassified designer opioids using molecular docking

Christopher R. Ellis, Naomi L. Kruhlak, Marlene T. Kim, E. Gregory Hawkins, Lidiya Stavitskaya

March 29, 2018

This document analyzes the raw data from the docking simulations, recreates the figures in the main text, and provides supporting information. The document can be reproduced by knitting 'SupportingInformation-OpioidDocking.Rmd' in Rstudio. It reads in data from:

1) opioid_docking_run1.txt
2) opioid_docking_run2.txt
3) opioid_docking_run3.txt
4) decoy_docking_data.txt
5) Fig5_data.txt

which contains the ouput from the opioid and decoy docking simulations. Specifically, data from 'opioid_docking_run1.txt' is used to create Figures 2-4 and 6-7 of the main text, and 'decoy_docking_data.txt' and 'Fig5_data.txt' are used for create Figure 7A, 7B and 7E from the main text. The files 'opioid_docking_run2.txt' and 'opioid_docking_run3.txt' are used to generate Supporting Information Figure 1 which demonstrates the reproducibility of the docking methodology.

In addition to this document, the supporting information package contains three directories. A description of the files included in each directory follows:

---

1) Decoys:

   - decoy_docking_output.mdb: database of the decoy-receptor complexes with scoring information from a decoy simulation.
   - decoys.P10000002.picked: Fentanyl decoy output from the DUD-E server.

---

2) Docking_structure:

   - mu_docking_structure.moe: mu opioid receptor structure used for docking.
   - mu_aminepharmacophore.ph4: pharmacophore used for initial docking placement.

- mu_docking_structure.pdb: coordinates of the prepared mu opioid receptor in PDB format (same structure as mu_docking_structure.moe).

---

3) MOE_and_PDB_complexes:

- drug-muor_complexes.mdb: MOE database that contains 24 representative opioid-muOR complexes with docking score.
- *opioid*-muor.pdb: Representative pdb structures for the 24 *opioid*-muOR complexes.

Table S1: Experimentally determined and predicted binding affinity of the 23 opioids docked at the μOR.

```
##                         ki means_data    sd_data
## alfentanil        7.39e+00 -10.510452 0.56230374
## buprenorphine     2.16e-01  -9.761342 0.48495542
## carfentanil       2.20e-01 -10.751093 0.30000160
## codeine           7.34e+02  -8.228554 0.15846246
## diphenoxylate     1.24e+01 -10.058808 0.46948775
## fentanyl          1.35e+00  -9.431270 0.38450949
## furanylfentanyl        NaN  -9.612408 0.33621356
## hydromorphone     3.65e-01  -7.941389 0.25599388
## lofentanil        5.50e-02 -10.809937 0.34086483
## Meperidine        4.50e+02  -7.773104 0.06892139
## morphine          1.14e+00  -7.757711 0.13668424
## nalbuphine        2.12e+00  -8.473987 0.28753606
## nmethylcarfentanil 4.20e+01  -8.532275 0.06820056
## nmethylfentanyl   1.80e+04  -7.891347 0.20712025
## oxycodone         2.59e+01  -8.781522 0.27128016
## oxymorphone       4.06e-01  -8.395338 0.18326080
## pentazocine       1.18e+02  -7.768322 0.12149920
## propoxyphene      1.20e+02  -9.560745 0.07504565
## R30490            9.00e-02 -10.258936 0.31000096
## Rmethadone        3.38e+00  -8.690919 0.11508095
## Rtramadol         1.25e+04  -7.848883 0.05840922
## Smethadone        3.38e+00  -8.631473 0.07020187
## Stramadol         1.25e+04  -7.967580 0.20757285
## sufentanil        1.38e-01  -9.892756 0.23320728
```

Fentanyl Derivatives

Table S2: Experimentally determined and predicted binding affinity of the 8 fentanyl derivatives docked at the μOR.

```
##                         ki means_data    sd_data
## nmethylfentanyl   1.80e+04  -7.891347 0.20712025
```

```
## nmethylcarfentanil 4.20e+01  -8.532275 0.06820056
## alfentanil         7.39e+00 -10.510452 0.56230374
## fentanyl           1.35e+00  -9.431270 0.38450949
## sufentanil         1.38e-01  -9.892756 0.23320728
## carfentanil        2.20e-01 -10.751093 0.30000160
## lofentanil         5.50e-02 -10.809937 0.34086483
## R30490             9.00e-02 -10.258936 0.31000096
```

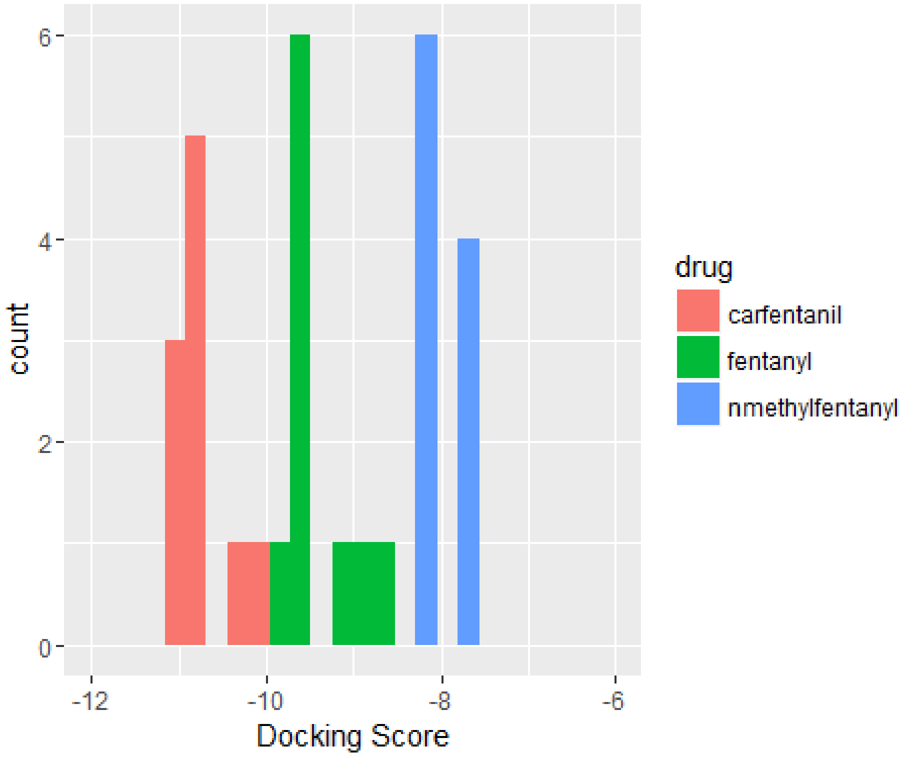

Main text Figure 2A: Docking score distribution from the 10 docking simulations of fentanyl (green), carfentanil (red) and N-methyl fentanyl (blue).

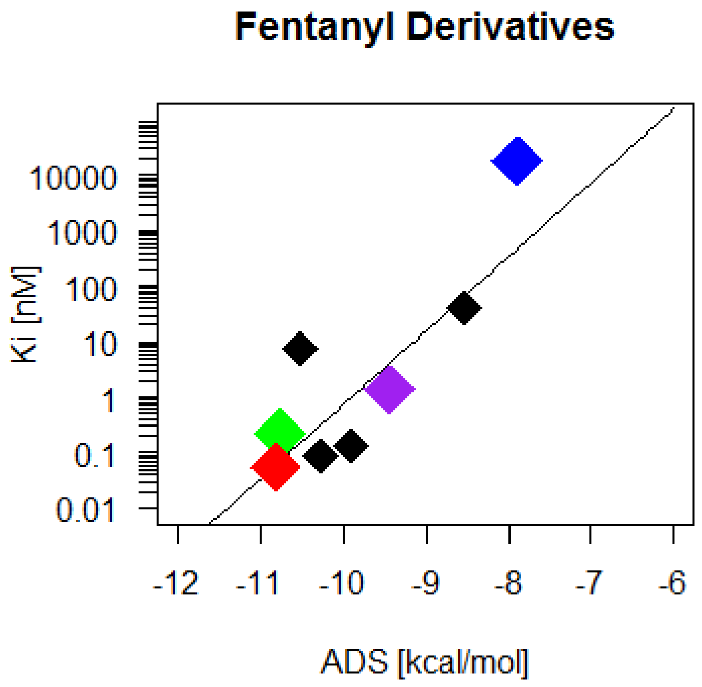

Main text Figure 3: Fentanyl analog binding prediction and classification. Scatterplot of the experimentally determined binding affinity, Ki, with the average docking score from the molecular docking procedure of the eight fentanyl analogs (shown as diamonds). The grey, shaded regions display the sub-nM, 1-100 nM, and greater than 100 nM binding concentration regimes used to classify the predicted binding strength of new drugs. N-methyl fentanyl (blue), fentanyl (purple), carfentanil (green) and lofentanil (red) are presented with colored diamonds to demonstrate the methods ability to separate fentanyl analogs into the proper binding concentration regimes. The remaining black diamonds represent the four other fentanyl analogs that were docked and scored.

Fentanyl Congeners

Table S3: Experimentally determined and predicted binding affinity of the 7 fentanyl congeners docked at the μOR.

```
##                     ki means_data    sd_data
## Rtramadol     12500.00   -7.848883 0.05840922
## Stramadol     12500.00   -7.967580 0.20757285
## Meperidine      450.00   -7.773104 0.06892139
## propoxyphene    120.00   -9.560745 0.07504565
## diphenoxylate    12.40 -10.058808 0.46948775
## Rmethadone        3.38   -8.690919 0.11508095
## Smethadone        3.38   -8.631473 0.07020187
```

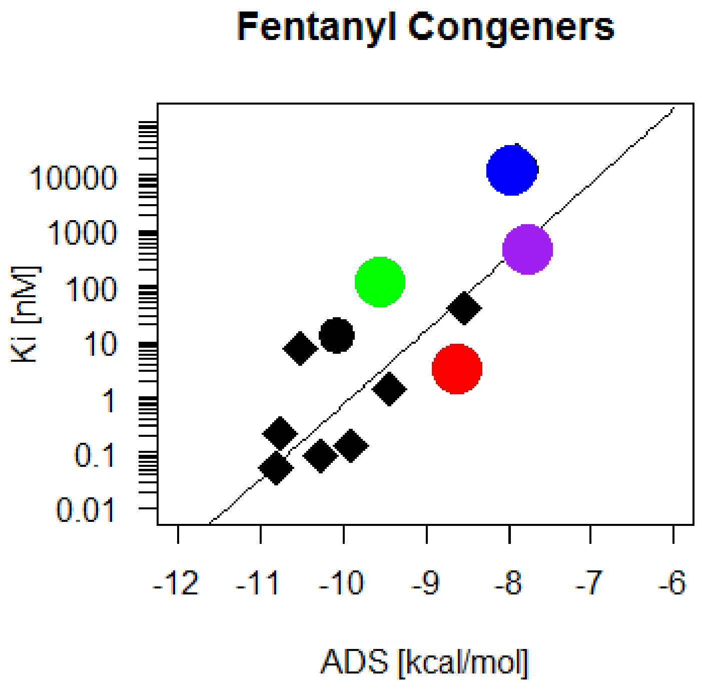

Main text Figure 4: Fentanyl congener binding prediction and classification. Scatterplot of the experimentally determined binding affinity, Ki, with the average docking score from the molecular docking procedure of the seven fentanyl congeners (shown as circles). (+)-Tramadol (blue), meperidine (purple), propoxyphene (green) and methadone (red) are highlighted and demonstrate the methods ability predict the correct binding concentration regimes of fentanyl congeners. The remaining black circles represent the three fentanyl congeners that were docked and scored. The black diamonds represent the fentanyl derivatives from Figure 3.

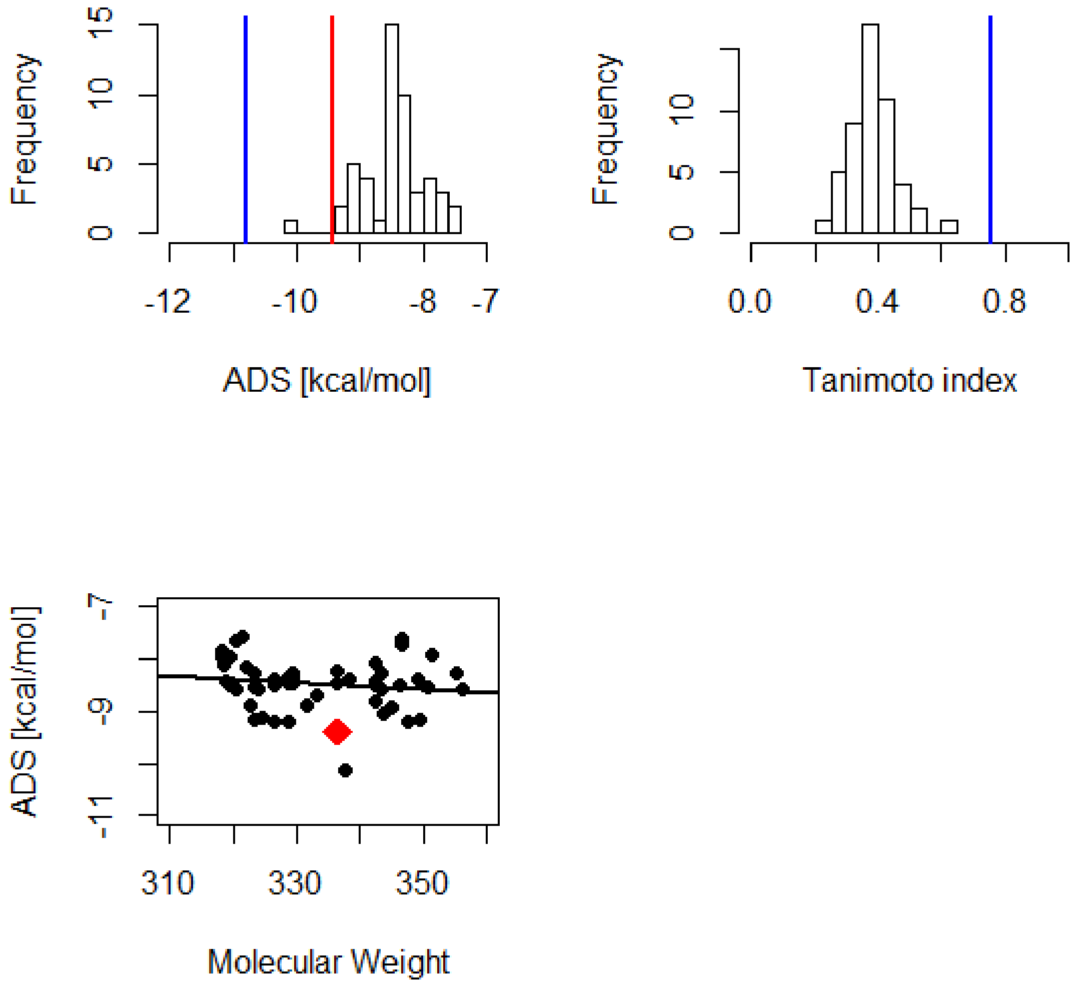

Main text Figure 5A, 5B, and 5E: : Decoy analysis. (A) Distribution of the decoy binding scores. The vertical red and blue lines indicate the docking score of fentanyl and carfentanil, respectively. (B) Distribution of the Tanimoto index of the fentanyl decoys with respect to fentanyl. The vertical blue line indicates the Tanimoto index of carfentanil (Tc = 0.76) with respect to fentanyl. (E) Correlation of the fentanyl decoy molecular weight with respect to the ADS. In panels C-E, the red and blue diamonds indicate fentanyl and carfentanil, respectively.

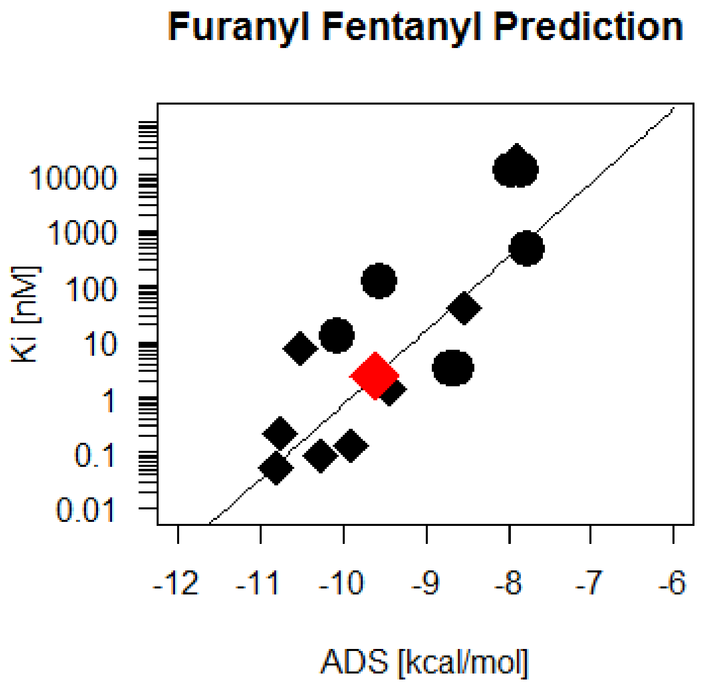

Main text Figure 6B: . Furanylfentanyl case study. (B) Binding concentration prediction of Fu-F (red).

Morphine Derivatives

Table S4: Experimentally determined and predicted binding affinity of the 8 morphine derivatives docked at the μOR.

```
##                     ki means_data   sd_data
## codeine        734.000  -8.228554 0.1584625
## pentazocine    118.000  -7.768322 0.1214992
## oxycodone       25.900  -8.781522 0.2712802
## nalbuphine       2.120  -8.473987 0.2875361
## morphine         1.140  -7.757711 0.1366842
## oxymorphone      0.406  -8.395338 0.1832608
## hydromorphone    0.365  -7.941389 0.2559939
## buprenorphine    0.216  -9.761342 0.4849554
```

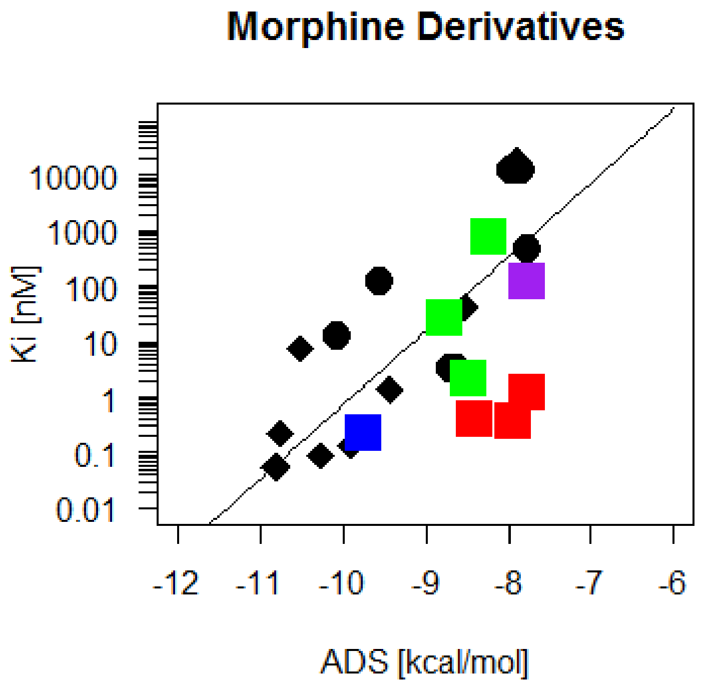

Maintext Figure 7: Morphine derivative binding prediction and classification. Scatterplot of the experimentally determined binding affinity, Ki, with the average docking score from the molecular docking procedure of the eight morphine derivatives (shown as squares). Pentazocine (purple) and buprenorphine (blue) are the most structurally dissimilar from morphine. The remaining six drugs are structurally similar to morphine; three are predicted correctly (green) and three are predicted incorrectly (red). The black diamonds and circles represent the fentanyl derivatives and fentanyl congeners from Fig 4.

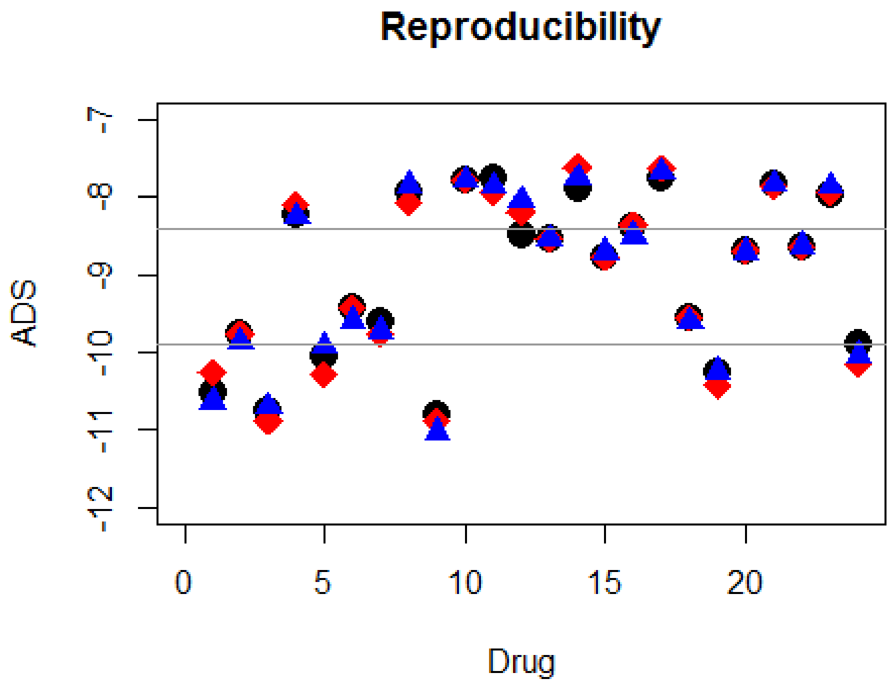

Supporting Information Figure 1: The docking procedure (ten independent simulations and averaging the best pose) was repeated three times for the 24 opioids. This plot shows the average docking score for run 1 (black), run 2 (red), and run 3 (blue).
